# Modest effect of p53, EGFR and HER-2/neu on prognosis in epithelial ovarian cancer: a meta-analysis

**DOI:** 10.1038/sj.bjc.6605112

**Published:** 2009-06-09

**Authors:** P de Graeff, A P G Crijns, S de Jong, M Boezen, W J Post, E G E de Vries, A G J van der Zee, G H de Bock

**Affiliations:** 1 Department of Gynaecologic Oncology, University Medical Centre Groningen, University of Groningen, Hanzeplein 1, Groningen 9713 GZ, The Netherlands; 2 Department of Medical Oncology, University Medical Centre Groningen, University of Groningen, Hanzeplein 1, Groningen 9713 GZ, The Netherlands; 3 Department of Epidemiology, University Medical Centre Groningen, University of Groningen, Hanzeplein 1, Groningen 9713 GZ, The Netherlands

**Keywords:** p53, EGFR, HER-2/neu, ovarian cancer, meta-analysis

## Abstract

**Background:**

P53, EGFR and HER-2/neu are the most frequently studied molecular biological parameters in epithelial ovarian cancer, but their prognostic impact is still unequivocal. We performed a meta-analysis to more precisely estimate their prognostic significance.

**Methods:**

Published studies that investigated the association between p53, EGFR and HER-2/neu status and survival were identified. Meta-analysis was performed using a DerSimonian–Laird model. Publication bias was investigated using funnel plots and sources of heterogeneity were identified using meta-regression analysis.

**Results:**

A total of 62 studies were included for p53, 15 for EGFR and 20 for HER-2/neu. P53, EGFR and HER-2/neu status had a modest effect on overall survival (pooled HR 1.47, 95% CI 1.33–1.61 for p53; HR 1.65, 95% CI 1.25–2.19 for EGFR and HR 1.67, 95% CI 1.34–2.08 for HER-2/neu). Meta-regression analysis for p53 showed that FIGO stage distribution influenced study outcome. For EGFR and HER-2/neu, considerable publication bias was present.

**Conclusions:**

Although p53, EGFR and HER-2/neu status modestly influences survival, these markers are, by themselves, unlikely to be useful as prognostic markers in clinical practice. Our study highlights the need for well-defined, prospective clinical trials and more complete reporting of results of prognostic factor studies.

Epithelial ovarian cancer is the leading cause of death from gynaecological cancers in the Western world. This high mortality is related to the difficulty to detect ovarian cancer at an early stage as well as the lack of effective therapies for advanced-stage disease (Cannistra, 2004).

Prognostic factors are defined as phenotypes, which correlate with the duration of (progression-free) survival (Agarwal and Kaye, 2005). In ovarian cancer, well-known clinicopathological prognostic factors in early-stage disease include differentiation grade and tumour rupture during surgery, whereas in late-stage disease histiotype, patient age, performance status and residual tumour after primary surgery are important prognostic factors (Vergote *et al*, 2001; Winter *et al*, 2007). Although these parameters do reflect biological features of both tumour and patient, they do not allow adequate prediction of outcome for the individual patient. The discovery of molecular biological prognostic factors should aid in a more accurate prediction of clinical outcome and may also reveal novel predictive factors and therapeutic targets (Oldenhuis *et al*, 2008).

The most frequently studied putative molecular biological prognostic factors in ovarian cancer are the tumour suppressor protein 53 (p53), and the oncogenes epidermal growth factor receptor (EGFR) and human epidermal growth factor receptor 2 (HER-2/neu). These markers also hold considerable promise as therapeutic targets. Agents targeting p53, EGFR and HER-2/neu proteins are currently under investigation in clinical trials (Dinh *et al*, 2008). However, evidence regarding their prognostic value with respect to survival is still inconclusive. Results of systematic reviews, including one from our institution, showed that these markers might predict prognosis in ovarian cancer, and also suggested considerable methodological variability (Crijns *et al*, 2003; Hall *et al*, 2004). The identification of these methodological weaknesses and sources of heterogeneity is important to improve the quality of future prognostic and predictive factor studies in ovarian cancer and other tumour types.

The aim of this study was to more precisely estimate the prognostic value of these markers and to adjust for methodological variability. We have used statistical methods developed by Parmar *et al* (1998) to indirectly estimate hazard ratios from Cox regression analyses and *P* values from log-rank tests, enabling us to incorporate a large number of studies in our meta-analyses. Moreover, we performed an in-depth analysis of study quality, the presence of publication bias and the extent and sources of heterogeneity between published studies.

## Materials and methods

### Search strategy and selection criteria

A MEDLINE, PubMed and EMBASE search for studies investigating the prognostic significance of p53, EGFR and HER-2/neu in ovarian cancer was performed. Studies published between 1990 and January 1st, 2009, were examined. MESH words used were ‘ovarian neoplasm’, ‘receptor epidermal growth factor’, ‘receptor erbB-2’ and ‘protein p53’. Additional words used for title search were marker^*^ or prognost^*^ or survival. The references of all publications and reviews were hand-searched to identify missing relevant publications.

Studies were included in the meta-analysis if they met the following criteria: (1) patients included had chemonaive epithelial ovarian cancer; (2) the endpoint investigated was disease specific or overall survival; (3) the study reported a hazard ratio (HR) and standard error (s.e.) or data sufficient to estimate the HR and s.e. from univariate survival analysis. Where a single study was reported on multiple occasions, only the report with the largest patient group or the most complete data was included. If a study reported results for more than one method (i.e., immunohistochemistry (IHC) and mutational analysis), for more than one well-described patient group or for multiple antibodies, results of all analyses were included in the meta-analysis. Thirteen studies published in languages other than English or German were excluded from the meta-analysis (for an overview, see Supplementary Table 1). Reviews, non-original articles and studies on non-epithelial or borderline ovarian tumours were also excluded.

**Figure 1:**
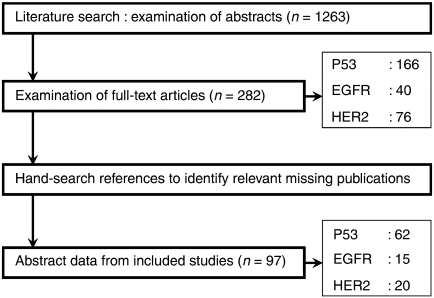
Search strategy.

Two researchers (PdG and APGC) independently examined abstracts of articles (*n*=614) to decide whether full-text articles should be obtained (Figure 1). Cases of disagreement were resolved by discussing the title and abstract. Full-text articles (*n*=216) were examined and excluded if a more detailed examination revealed that they did not meet the inclusion criteria. The sample size of included studies did not differ from the sample size of excluded studies (data not shown). Where applicable, we adhered to the QUORUM criteria for improving the quality of reporting of meta-analyses (Moher *et al*, 1999).

### Data extraction

Data were extracted independently by two investigators (PdG and APGC) by means of a predefined form. Topics in this form were year of publication, country, number of patients, years of patient inclusion, method of case selection (retrospective or prospective cohort of patients), age at time of diagnosis (mean, median, range), distribution of stage, tumour type and differentiation grade, treatment, amount of residual tumour after primary surgery, response to chemotherapy, time of follow-up (median, mean, minimum and maximum), assay method and scoring protocol used, number of marker positive and negative tumours, numbers of (disease specific and overall) death, and results of univariate survival analyses.

### Assessment of study quality and publication bias

Study quality was assessed independently by two investigators (PdG and APGC) by means of a predefined form. As there are no generally accepted standards for measuring study quality, this form was derived from the work of McShane *et al* (2005) and Hayes *et al* (1996) (Supplementary Table 2). In summary, the following criteria were investigated: whether (1) the study reported inclusion and exclusion criteria; (2) study data were prospectively or retrospectively gathered; (3) patient and tumour characteristics were sufficiently described; (4) the assay used to measure biomarker expression was sufficiently described; (5) a definition of the study endpoint was provided; (6) the follow-up time of patients in the study was described; (7) the study reported how many patients were lost to follow-up or were not available for statistical analysis. Studies with a total score of 8 were considered to show the highest study quality, whereas a zero score indicated the lowest quality.

Additionally, studies were scored as phase I–III prognostic marker studies according to the classification proposed by Simon and Altman (1994). Early exploratory studies are designated phase I studies, whereas phase II studies investigate the association of a biomarker with patient prognosis and are hypothesis generating in nature, and phase III studies are large confirmatory studies of prestated hypotheses.

Publication and selection bias were investigated through a funnel plot (Egger *et al*, 1997).

### Statistical analysis

Statistical analyses were carried out using SPSS version 12.01 (SPSS, Chicago, IL, USA), Review Manager version 4.2 (The Cochrane Collaboration, the Nordic Cochrane Centre, Copenhagen, Denmark) and MLWIN version 2.0 (Centre for Multilevel Modelling, University of Bristol, Bristol, UK).

The first goal of our meta-analysis was to obtain a log-hazard ratio and its standard error for each study according to methods previously described by Parmar *et al* (1998). If the study reported results of a univariate Cox regression analysis, log-hazard and its standard error were directly included in the meta-analysis. When the study did not report the standard error, it was estimated from the 95% confidence interval (CI) or *P* value of univariate Cox regression analyses. If results of univariate Cox regression analyses were not presented in the paper, the log-hazard ratio and its standard error were estimated indirectly from *P* values of the log-rank test. Subsequently we performed a meta-analysis using the DerSimonian–Laird random effects model (DerSimonian and Laird, 1986), applying the inverse of variance as a weighing factor. Heterogeneity was investigated by use of the *I*^2^ statistic, which takes values from 0 to 100% (Higgins and Thompson, 2002). An *I*^2^ value >50% was considered to represent substantial heterogeneity between studies.

Quantitative assessment of sources of heterogeneity was undertaken by meta-regression analysis (Thompson and Higgins, 2002). The following potential sources of heterogeneity were explored: study quality score, year of publication (< or > median year of publication), data collection (prospective or retrospective), region (Europe, United States, Asia or other), FIGO stage (< or >50% FIGO stage III/IV tumours), tumour type (<50% or >50% serous tumours), differentiation grade (<50% or >50% grade III or undifferentiated tumours), type of tumour tissue (frozen or paraffin-embedded), assay method (IHC or other), primary antibody (monoclonal or polyclonal), cut-off value for positive marker expression (< or > median percentage of positive tumours) and percentage of positive tumours (< or > number of percentage positive tumours). For each potential source of heterogeneity, a multilevel model was developed with the logHR as dependent variable and the sources of heterogeneity as independent variables.

## Results

### Study characteristics

For p53, 62 studies reporting results of 75 analyses in 9448 patients were included (Supplementary Table 3; median study size 102 patients, range 20–783; Hartmann *et al*, 1994; Klemi *et al*, 1995; Allan *et al*, 1996; Eltabbakh *et al*, 1997; Viale *et al*, 1997; Darai *et al*, 1998; Marx *et al*, 1998; Silvestrini *et al*, 1998; Anttila *et al*, 1999; Baekelandt *et al*, 1999; Kassim *et al*, 1999; Wen *et al*, 1999; Blegen *et al*, 2000; Laframboise *et al*, 2000; Levesque *et al*, 2000; Ozalp *et al*, 2000; Schildkraut *et al*, 2000; Shahin *et al*, 2000; Birner *et al*, 2001; Howells *et al*, 2001; Reles *et al*, 2001; Saegusa *et al*, 2001; Schuyer *et al*, 2001; Skirnisdottir *et al*, 2001a; Berker *et al*, 2002; Hawes *et al*, 2002; Pieretti *et al*, 2002; Sagarra *et al*, 2002; Havrilesky *et al*, 2003; Ikeda *et al*, 2003; Konstantinidou *et al*, 2003; Nakayama *et al*, 2003; Tachibana *et al*, 2003; Wisman *et al*, 2003; Bali *et al*, 2004; Ceccaroni *et al*, 2004; Iba *et al*, 2004; Nielsen *et al*, 2004; Seo *et al*, 2004; Concin *et al*, 2005; Goodheart *et al*, 2005; Kaern *et al*, 2005; Kaiser *et al*, 2005; Terauchi *et al*, 2005; Green *et al*, 2006; Lee *et al*, 2006; Ueno *et al*, 2006; Yakirevich *et al*, 2006; de Graeff *et al*, 2006; Brustmann, 2007; Galic *et al*, 2007; Malamou-Mitsi *et al*, 2007; Materna *et al*, 2007; Psyrri *et al*, 2007; Bartel *et al*, 2008; Darcy *et al*, 2008; Garcia-Velasco *et al*, 2008; Giordano *et al*, 2008; Kobel *et al*, 2008; Leffers *et al*, 2008; Tomsova *et al*, 2008; Vartiainen *et al*, 2008). There were 13 prospective studies and 49 retrospective studies. All studies were designated phase II biomarker studies. No phase III biomarker studies were found, although two large studies fulfilled almost all requirements (de Graeff *et al*, 2006; Darcy *et al*, 2008). Most studies used IHC (*n*=60) or mutational analysis (single-strand conformation polymorphism analysis and/or sequencing, *n*=11) to determine p53 status. Other methods included fluorescence *in situ* hybridisation (FISH, *n*=1) and immunoassays (*n*=2). For IHC staining, the most frequently used antibodies were DO1 (*n*=10) and DO7 (*n*=32). Six studies did not specify the antibody used. Cut-off values for positive immunostaining varied widely, ranging from >5% to >90% nuclear staining. The median percentage of p53 positive tumours was 50% (range 13.7–82.0%). A significant association of p53 expression with overall survival in univariate analysis was reported in 29 (38.6%) analyses, of which 25 reported an association with poor survival and 4 an association with improved survival.

For EGFR, 15 studies in 2471 patients were included in the meta-analysis (Supplementary Table 4; median study size 106 patients, range 40–783; Kaufmann *et al*, 1995; Scambia *et al*, 1995; Bartlett *et al*, 1996; Fischer-Colbrie *et al*, 1997; Skirnisdottir *et al*, 2001b; Elie *et al*, 2004; Nielsen *et al*, 2004; Psyrri *et al*, 2005; Raspollini *et al*, 2005; Schilder *et al*, 2005; Wang *et al*, 2005; Castellvi *et al*, 2006; Lassus *et al*, 2006; Brustmann, 2008; de Graeff *et al*, 2008). Again, all studies were classified as phase II biomarker studies. Three studies prospectively collected data. Eleven studies performed IHC staining for determination of EGFR expression using five different antibodies and six cut-off values for positive EGFR expression. Other methods included ^125^EGF binding assay (*n*=3) and RT–PCR (*n*=1). Positive immunostaining was observed in 6.2–72.6% (median 35%) of tumours, and in 7 studies (63.6%) EGFR expression predicted poor overall survival.

For HER-2/neu, 20 studies reporting results of 21 analyses in 3055 patients were subjected to final analysis (Supplementary Table 4; median study size 111 patients, range 40–783; Berchuck *et al*, 1990; Fajac *et al*, 1995; Kaufmann *et al*, 1995; Medl *et al*, 1995; Wang *et al*, 1999, 2005; Davidson *et al*, 2000; Skirnisdottir *et al*, 2001b; Camilleri-Broet *et al*, 2004; Nielsen *et al*, 2004; Verri *et al*, 2005; Castellvi *et al*, 2006; Surowiak *et al*, 2006; Malamou-Mitsi *et al*, 2007; Pils *et al*, 2007; Steffensen *et al*, 2007; Tuefferd *et al*, 2007; de Graeff *et al*, 2008; Garcia-Velasco *et al*, 2008; Tomsova *et al*, 2008). All studies were designated phase II biomarker studies. Two studies prospectively collected patient data. Methods to determine HER-2/neu status included IHC (*n*=16) with 3 studies additionally performing FISH for ambiguous cases, PCR (*n*=1), FISH only (*n*=1), Southern blot (*n*=1) and HER-2/neu immunoassay (*n*=1). Antibodies used for IHC staining included CB11 (*n*=3), TA1 (*n*=1), MCO102 (*n*=1), NCL-CBE-356 (*n*=1), the Herceptest kit (*n*=4) and unspecified antibodies (*n*=3). Five different cut-off values for positive HER-2/neu protein expression were used. The median percentage of positive tumours was 18.0% (range 5–57%). Eight studies (40%) reported that HER-2/neu was a significant predictor of overall survival in univariate analysis, of which one study reported an association between HER-2/neu staining and improved survival.

### Quality assessment and publication bias

The median quality score was 5 (range 1–8) for p53, 5 for EGFR (range 3–7) and 5 for HER-2/neu (range 3–8; Supplementary Tables 3–5). High study quality was related to a high journal impact factor for p53 (*P*=0.010), but not for EGFR (*P*=0.59) and HER-2/neu (*P*=0.65).

**Figure 2:**
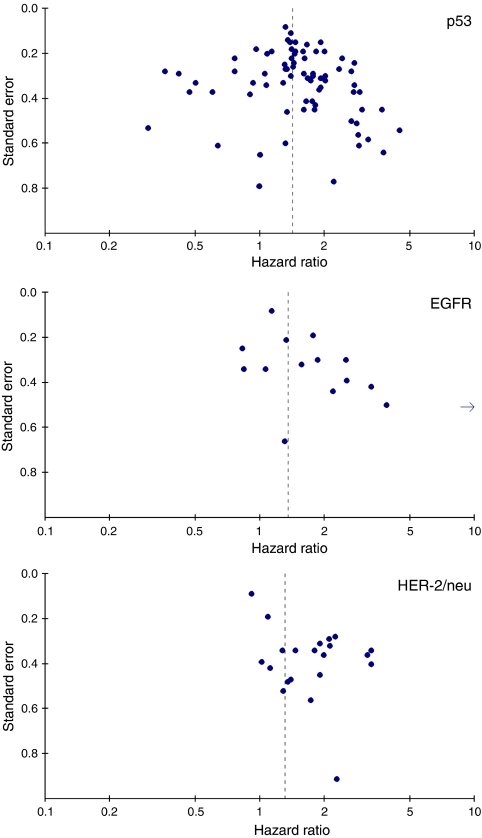
Funnel plots. Funnel plots showing the relationship between the effect size of individual studies (hazard ratios for overall survival, horizontal axis) and the precision of the study estimate (standard error, vertical axis) for p53, EGFR and HER-2/neu.

Investigation of bias by a funnel plot showed substantial funnel plot asymmetry for HER-2/neu and EGFR, suggesting the presence of publication and/or selection bias (Figure 2). For p53, no funnel plot asymmetry was found.

### Meta-analysis and assessment of heterogeneity

#### P53

**Figure 3:**
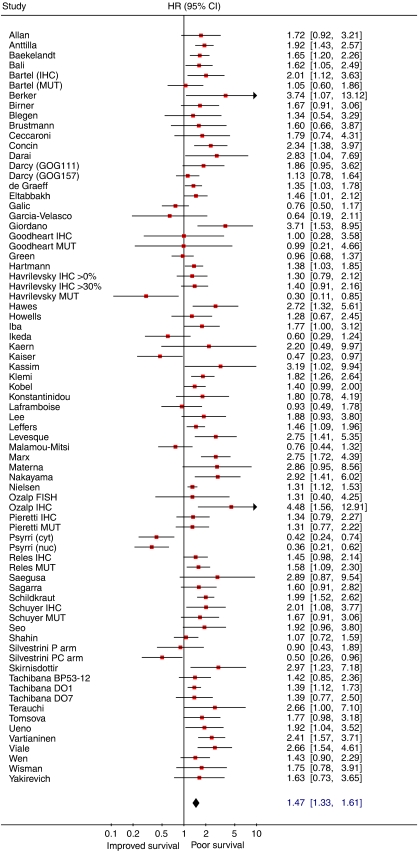
Forest plot showing results of studies on the prognostic value of p53 expression. Hazard ratios and 95% CI (confidence interval) of individual studies for patients with p53 positive tumours. Hazard ratios: squares whose heights are inversely proportional to the standard error of the estimate, and their respective confidence intervals (horizontal lines). Summary hazard ratio: diamond with horizontal limits at the confidence limits and width inversely related to its standard error. Hazard ratios higher than 1 indicate an increased risk of death for patients with a tumour with aberrant p53 status. Abbreviations: MUT=results of mutation analysis; IHC=results of immunohistochemical staining; cyt=results for cytoplasmic immunostaining; nucl=results for nuclear immunostaining; P arm=results for patients treated with cisplatin; PC arm=results for patients treated with cisplatin/cyclophosphamide.

Meta-analysis of 53 studies on the prognostic value of p53 expression showed that aberrant of p53 status is associated with poor overall survival (HR obtained from DerSimonian–Laird random effects model: 1.55 (95% CI 1.40–1.71); Figure 3), although there was heterogeneity between studies (*I*^2^=44.4%). Subgroup analysis revealed a prognostic impact for IHC studies, IHC studies with the DO7 antibody, studies using mutational analysis and studies with a quality score >6. However, considerable heterogeneity remained present, indicating that not all sources of heterogeneity could be accounted for (Table 1). When the meta-analysis was restricted to studies reporting results of (subgroup) analyses for serous tumours (Bali *et al*, 2004; Terauchi *et al*, 2005; Ueno *et al*, 2006; Yakirevich *et al*, 2006; Kobel *et al*, 2008; Vartiainen *et al*, 2008) p53 status was also a predictor of poor survival. Unfortunately, the number of studies reporting results for the other histological subtypes was too small to perform a pooled analysis. Meta-regression analysis revealed that the outcome of analysis was influenced by FIGO stage distribution. When results of six studies reporting results for stage III/IV tumours were subsequently pooled, p53 status was no longer of prognostic value (Table 1).

#### EGFR

**Figure 4:**
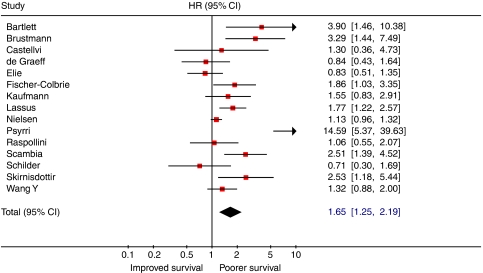
Forest plot showing results of studies on the prognostic value of EGFR expression. Hazard ratios and 95% confidence intervals for patients with EGFR positive tumours (symbols as in Figure 3).

Results of meta-analysis for EGFR showed a significant relationship between overexpression of EGFR and poor patient outcome (HR: 1.65 (95% CI 1.25–2.19); Figure 4). Although significant heterogeneity was present (*I*^2^=74.3%), the sources of heterogeneity could not be determined in meta-regression analysis. Restricting the analysis to studies that used IHC staining for determination of marker expression did not alter results of heterogeneity tests (Table 1). However, further analysis showed that heterogeneity was partly due to results of the study by Psyrri *et al* (2005). When this study was excluded from the meta-analysis, less heterogeneity was observed.

#### HER-2/neu

**Figure 5:**
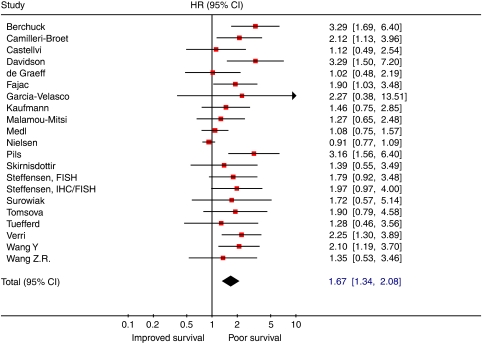
Forest plot showing results of studies on the prognostic value of HER-2/neu expression. Hazard ratios and 95% CI (confidence intervals) for patients with HER-2/neu positive tumours (symbols as in Figure 3).

Meta-analysis of univariate analyses on the prognostic value of HER-2/neu showed that overexpression of HER-2/neu is associated with poor overall survival (HR: 1.67 (95% CI 1.34–2.08); Figure 5), but again considerable heterogeneity was present (*I*^2^=59.6%). Of note, none of the studies using immunohistochemical staining followed by FISH for ambiguous samples reported a statistically significant relationship between HER-2/neu expression and survival (Castellvi *et al*, 2006; Malamou-Mitsi *et al*, 2007; Tuefferd *et al*, 2007). The most important factor explaining the lack of homogeneity between studies was study quality, with studies of low quality reporting more significant results.

## Discussion

In this study, we present a pooled estimate of the prognostic value of p53, EGFR and HER-2/neu in epithelial ovarian cancer. Our results show that as single markers, p53, EGFR and HER-2/neu are not likely to be useful as prognostic factors in clinical practice (pooled HR for all included studies: 1.47 (95% CI 1.33–1.61) for p53; 1.65 (95% CI 1.25–2.19) for EGFR and 1.67 (95% CI 1.34–2.08) for HER-2/neu). Furthermore, our study clearly indicates that adequate conduct and complete reporting are imperative for improving the quality of prognostic factor studies in ovarian cancer.

Although protein expression of p53 and EGFR as assessed by IHC staining has a modest effect on prognosis, neither p53 nor EGFR immunostaining predicts clinical outcome in a manner comparable to well-known clinicopathological prognostic factors such as tumour stage and residual tumour after primary surgery. Our results also show that p53 mutations have prognostic value in epithelial ovarian cancer, although this was of borderline significance. However, this analysis was affected by small sample size and methodological issues, such as the use of different techniques for mutational analyses and the analysis of different exons.

For HER-2/neu and EGFR, the ability to draw reliable conclusions from meta-analysis was affected by the presence of considerable publication bias for studies with a small sample size yielding non-significant results. The presented hazard ratios might, therefore, be an overestimation of the true effect size. More importantly, meta-regression analysis demonstrated that studies that are poorly designed or reported produce higher estimates of the prognostic value of HER-2/neu. This finding has previously been demonstrated in a meta-analysis of clinical trials, where incorporation of results of poor quality randomised controlled trials contributed to significant exaggeration of treatment efficacy (Moher *et al*, 1998).

It has long been appreciated that the histological subtypes of ovarian cancer show considerable differences with respect to stage at diagnosis, response to chemotherapy and underlying molecular abnormalities (Bell, 2005). This was recently demonstrated by Kobel *et al* (2008), who assessed the expression of 21 candidate biomarkers in a large cohort of 500 ovarian carcinomas and subsequently performed subgroup analyses for the different histological subtypes. Their results showed that the expression as well as the prognostic value of most biomarkers considerably varied between the subtypes. In this study, we assessed the prognostic value of p53 in six studies presenting (subgroup) analyses for p53 in serous tumours. The results of this analysis did not show a large difference between the prognostic value of p53 in serous tumours and it prognostic value in the entire cohort. Additionally, we performed a subgroup analysis for four studies reporting six analyses on the prognostic value of p53 in stage III/IV tumours. In this group, p53 was not of prognostic value. However, the number of studies that could be analysed was small and we were not able to perform a pooled analysis for the other histological subtypes. Our results underscore the importance of biomarker analysis in homogeneous subgroups of patients, such as patients with a particular disease stage, tumour type or differentiation grade. To perform these kinds of analyses, international collaboration is critical. Furthermore, the submission of raw, uncategorised study data to public databases would allow for analysis of specific subgroups although maintaining prognostic power.

Most studies in the meta-analysis used IHC staining to study expressions of p53, EGFR and HER-2/neu. Although IHC staining is simple and cost-effective to perform, results are highly dependent on a variety of methodological factors such as storage time and fixation method of paraffin-embedded tissues, choice of primary antibody and IHC staining protocol (Jacquemier *et al*, 1994; Hall *et al*, 2004). In this study, differences in IHC staining protocols and cut-off values for positive protein expression ranging from >5 to >90% positively stained cells may have contributed to the observed heterogeneity. Our results, therefore, make a strong case for international consensus on staining and scoring protocols.

As a first step towards quality assessment of prognostic factor studies to be included in meta-analyses, we have developed a quality score. For meta-analyses evaluating results of both clinical trials and diagnostic studies, such criteria are available and are widely used to either exclude studies low-quality studies or evaluate study quality (Jadad *et al*, 1996; Whiting *et al*, 2003). As our quality score was newly developed for this study and was not extensively validated, we chose not to exclude studies from statistical analysis beforehand because of a low score. Based on results of meta-regression analysis we do, however, believe that it provides a good estimation of study quality. In future studies, our quality score might serve as a further step towards the development of evidence-based quality assessment tools for meta-analyses of prognostic factor studies. In addition, the use of the recently published REMARK guidelines for reporting of prognostic factor studies will aid in a more complete and transparent reporting (McShane *et al*, 2005), thereby also increasing the number of high-quality studies that can be included in a meta-analysis.

We have also designated all studies phase I–III prognostic factor studies according to a classification proposed by Simon and Altman (1994). Although several large studies on the prognostic value of p53 and HER-2/neu have been performed, no studies met the stringent criteria for phase III biomarker studies. A prespecified hypothesis, the description of eligibility criteria and a sufficiently large number of patients were often lacking. In addition, almost none of the studies were specifically designed to determine the prognostic impact of p53, EGFR or HER-2/neu as single markers. These results underscore the need for well-designed studies with clearly stated hypotheses that examine the relationship between biomarker expression and clinical outcome.

Although this study shows that p53, EGFR and HER-2/neu immunostaining do not have a strong direct relationship with survival, it is more than likely that their respective pathways do influence patient prognosis. In future studies, several approaches could be taken to elucidate the prognostic value of these pathways. For instance, IHC staining of activated (phosphorylated) receptors and key regulatory proteins involved in upstream and downstream signalling may be more informative than immunostaining of single markers regardless of their activation status (Wang *et al*, 2005; de Graeff *et al*, 2008). In addition, other methods to assess pathway activation status may be employed to identify prognostic factors. For instance, EGFR amplification as determined by FISH has been shown to be independently associated with poor survival in vulvar cancer and in head and neck squamous cell carcinomas (Chung *et al*, 2006; Growdon *et al*, 2008). Two recent reports in ovarian cancer also suggest that increased gene copy number of EGFR is more strongly related to survival than protein expression (Lassus *et al*, 2004, 2006).

Other attractive approaches for the identification of novel prognostic and predictive factors include the identification of genes and pathways by microarray analysis. Traditional prognostic factor studies, including those on p53, HER-2/neu and EGFR, have until now mainly focused on the prognostic value of single genes. Over the past years, it has become apparent that this ‘one gene, one outcome’ hypothesis is an oversimplification of the multiple genetic and epigenetic mechanisms that account for ovarian cancer survival. Using pathway analysis of large datasets such as microarray data (Bild *et al*, 2006), alterations in the p53, EGFR and HER-2/neu pathways rather than single genes can be analysed. Ultimately, the identification or deregulated pathways in a single tumour may lead to a more precise estimation of patient prognosis and might also reveal novel therapeutic targets. However, these studies often need a far more complex design and statistical analysis compared to single marker studies. It is, therefore, especially important to address methodological issues when designing and reporting these analyses, and to take possible sources of heterogeneity into account.

There are some limitations to this meta-analysis. Firstly, especially for EGFR and HER-2/neu considerable heterogeneity was observed. When subgroup analyses for more homogeneous groups of studies was performed, for example, only studies performing IHC staining, heterogeneity remained present. This indicates that not all sources of heterogeneity could be accounted for in this meta-analysis, and that results should be interpreted with caution. Secondly, we have restricted our analysis to published studies written in English or German. Thirteen, mostly small studies that met eligibility criteria according to the abstracts were excluded based on language criteria. This may result in publication or language bias leading to an overestimation of effect sizes (Egger *et al*, 1997; Pham *et al*, 2005). Although this was not the case for p53, there was clear evidence of publication bias for EGFR and HER-2/neu. Thirdly, our meta-analysis is based on unadjusted estimates, whereas a more precise estimate could be obtained using a multivariate analysis adjusting for clinicopathological variables. However, multivariate analyses reported in the included studies used various models and different covariates, and could, therefore, not be combined into a pooled estimate.

In conclusion, our study shows that although aberrations of p53 and EGFR have a modest effect on survival in ovarian cancer, they are currently unlikely to influence clinical decision-making. Identification of multiple methodological flaws and sources of heterogeneity in currently available prognostic factor studies should contribute to improve design and reporting of future prognostic and predictive factor studies. Hopefully, this way, deregulated molecular biological factors/pathways will be identified that will make a difference in clinical decision making, ultimately resulting in effective, individualised targeted therapy for ovarian cancer patients.

## Figures and Tables

**Table 1:** Summarized hazard ratios

**Analysis**	***N***	**Pooled HR (95% CI)^a^**	***I*^2^ value (%)**	*P*-value*
*P53*				
All studies	75	1.47 (1.33–1.61)	58.9	<0.001
Studies using IHC staining	41	1.47 (1.33–1.64)	59.8	<0.001
Studies using IHC staining with the DO7 antibody	32	1.49 (1.26–1.75)	69.0	<0.001
Studies using mutational analysis	11	1.33 (1.03–1.70)	47.7	0.03
Studies with a quality score ⩾5^b^	40	1.44 (1.27–1.63)	66.4	<0.001
Studies restricted to serous tumours^c^	6	1.61 (1.09–2.38)	61.3	0.02
Studies restricted to stage III/IV tumours	8	0.91 (0.59–1.39)	71.8	<0.001
				
*HER-2/neu*				
All studies	21	1.67 (1.34–2.08)	59.8	<0.001
Studies using IHC staining	13	1.78 (1.28–2.46)	73.0	<0.001
Studies with a quality score ⩾5^b^	12	1.46 (1.13–1.89)	57.0	0.008
				
*EGFR*				
All studies	15	1.65 (1.25–2.19)	74.3	<0.001
Studies using IHC staining	11	1.50 (1.08–2.09)	76.6	<0.001
All studies except Psyrri *et al* (2005)	14	1.47 (1.17–1.84)	59.5	0.002

HR=hazard ratio; 95% CI=95% confidence interval; IHC=immunohistochemical staining.

a Pooled hazards ratios were obtained from using a DerSimonian–Laird random effects model, applying the inverse of variance as a weighing factor.

b Cut-off values for quality scores were based on the median quality score of included studies for a specific marker.

c Four studies restricted to serous tumours and two studies (Ueno *et al*, 2006; Kobel *et al*, 2008) reporting results of subgroup analysis for serous tumours.

^*^P values obtained from *χ*^2^-test for heterogeneity.
