# Supplementary material for: Modest effect of p53, EGFR and HER-2/neu on prognosis in epithelial ovarian cancer: a meta-analysis
Source: Br J Cancer. 2009 Jun 9;101(1):149–59. doi: 10.1038/sj.bjc.6605112 (PMC2713689; doi:10.1038/sj.bjc.6605112)
Supplement: Supplementary Table 1 [file 6605112x1.doc]

## Supplementary table 1: Overview of studies that were excluded based on language criteria*

| *Reference* | *Journal* | *Year of publication* | *Sample size* | *Markers under study* |
| --- | --- | --- | --- | --- |
| [Bar et al., 2002] | Ginekol Pol | 2002 | 49 | P53 |
| [Coronado Martin et al., 2007] | Med Clin (Barc). | 2007 | 124 | P53, HER-2/neu |
| [Frutuoso et al., 2001] | Acta Med Port | 2001 | 81 | HER-2/neu |
| [Furugen, 1991] | Nippon Sanka Fujinka Gakkai Zasshi | 1991 | ? | EGFR |
| [Li et al., 2002] | Ai Zheng | 2002 | 84 | HER-2/neu |
| [Liu and Yang, 1999] | Zhonghua Fu Chan Ke Za Zhi | 1999 | ? | HER-2/neu |
| [Nakano et al., 1998] | Gan To Kagaku Ryoho | 1998 | 31 | P53 |
| [Sakamoto, 1999] | Acta Obstet Gynaecol Jpn | 1999 | 62 | P53, HER-2/neu |
| [Stepanova et al., 2005] | Vopr Onkol | 2005 | ? | EGFR |
| [Tomov et al., 2007] | Akush Ginekol | 2007 | ? | EGFR |
| [Xin, 1993] | Zhongua Fu Chan Ke Za Zhi | 1993 | 17 | HER-2/neu |
| [Yu M. et al., 2005] | Chin J Clin Oncol | 2005 | 50 | P53, HER-2/neu |
| [Zhang et al., 2008] | Ai Zheng | 2008 | 76 | EGFR |

* Studies on the prognostic value of p53, HER-2/neu and EGFR for which the full-text articles were not obtained based on language criteria

Reference List

Bar JK, Sobanska E, Popiela A, Goluda M (2002) [Evaluation of expression and correlation between P53, BCL-2 and BAX proteins in ovarian neoplasms]. *Ginekol Pol* **73**: 102-109

Coronado Martin PJ, Fasero Laiz M, Garcia Santos J, Ramirez Mena M, Vidart Aragon JA (2007) [Overexpression and prognostic value of p53 and HER2/neu proteins in benign ovarian tissue and in ovarian cancer]. *Med Clin (Barc )* **128**: 1-6

Frutuoso C, Silva MR, Amaral N, Martins I, De Oliveira C, De Oliveira HM (2001) [Prognosis value of p53, C-erB-2 and Ki67 proteins in ovarian carcinoma]. *Acta Med Port* **14**: 277-283

Furugen Y (1991) [Immunohistochemical studies on epidermal growth factor receptor (EGF-R) in gynecological malignant tumor]. *Nippon Sanka Fujinka Gakkai Zasshi* **43**: 410-416

Li JD, Li MD, Li YF, Huang X, Liu JH, Liu FY, Zhang CQ (2002) [Relationship between expressions of p53, c-erbB2 genes, proliferating cell nuclear antigen and prognosis of patients with ovarian epithelial carcinoma]. *Ai Zheng* **21**: 292-296

Liu L, Yang K (1999) [A study on C-erbB2, nm23 and p53 expressions in epithelial ovarian cancer and their clinical significance]. *Zhonghua Fu Chan Ke Za Zhi* **34**: 101-104

Nakano T, Enoki K, Nakashima M, Ishikawa H, Ametani Y, Ohta S, Ohkuchi A, Satake S, Kojima Y, Funamoto H, Tateno M, Miwa A (1998) [Survival in patients with clear cell carcinoma of the ovary]. *Gan To Kagaku Ryoho* **25**: 67-73

Sakamoto H (1999) Molecular biology of multidrug resistance (MDR) in ovarian cancers and novel method of detecting developing MDR in vitro. *Acta Obstetrica et Gynaecologica Japonica* **51**: 549-561

Stepanova EV, Polushkina IN, Perevoshchikov AA, Ermilova VD, Vishnevskaia I, Meshcheriakov AA, Baryshnikov AI, Lichinitzer MR (2005) [Expression of epidermal growth factor receptor (EGFR) in ovarian carcinoma stage III-IV]. *Vopr Onkol* **51**: 361-365

Tomov S, Gorchev G, Khinkova N, Tsingilev D (2007) [Clinical aspects in expression of epidermal growth factor receptors in epithelial ovarian tumors]. *Akush Ginekol (Sofiia)* **46**: 29-33

Xin XY (1993) [The amplification of c-myc, N-ras, c-erb B oncogenes in ovarian malignancies]. *Zhonghua Fu Chan Ke Za Zhi* **28**: 405-7, 442

Yu M., Hao J., Jiao Z (2005) A study on the expression of BRCA1 & P53 and their correlation in epithelial ovarian cancer. *Chin J Clin Oncol* **32**: 18-20

Zhang J, Chen AP, Wang B, Zhao SP, Liu LZ, Dai SZ (2008) [Correlations of EGFR and LRP to chemotherapy resistance and prognosis of ovarian cancer]. *Ai Zheng* **27**: 1331-1336
