# Supplementary material for: Modest effect of p53, EGFR and HER-2/neu on prognosis in epithelial ovarian cancer: a meta-analysis
Source: Br J Cancer. 2009 Jun 9;101(1):149–59. doi: 10.1038/sj.bjc.6605112 (PMC2713689; doi:10.1038/sj.bjc.6605112)
Supplement: Supplementary Table 2 [file 6605112x2.doc]

## Supplementary table 2: Criteria for quality assessment

**Criterium: ___Points**

1. Is the population under study defined with in- and exclusion criteria? 1

2. Were patient data prospectively collected? 1

3. Are the main prognostic patient and tumour characteristics presented?^1^ 1

4. Is the method used for determination of protein expression specified? 2

- 4.1. Criteria for immuhistochemistry / FISH:
  - Is the immunohistochemical staining protocol specified?^2^ 1
  - Were stainings evaluated by > 1 observer? 1
- 4.2. Criteria for mutational analysis:
  - Is the PCR protocol specified?^3^ 1
  - Is the SSCP and/or sequencing protocol specified? 1
- 4.3. Criteria for Southern Blot:
  - Are the restriction enzymes used specified? 1
  - Is the hybridization methods specified?^4^ 1
- 4.4. Criteria for EGF binding assay:
  - Are positive and negative controls specified? 1
  - Is the assay protocol specified?^5^ 1
- 4.5. Criteria for RT-PCR:
  - Is the RNA isolation method and cDNA synthesis specified? 1
  - Is the PCR protocol specified?^3^ 1
- 4.6. Criteria for enzyme immunoassay
  - Is the antibody used specified? 1
  - Are control samples and a cut-off value for positive expression specified? 1

5. Is the study endpoint defined? 1

6. Is the time of follow up specified? 1

7. Is loss during analysis or follow up described? 1

*-----------------------*

Max. 8 points

1) At least four of the following characteristics: age at diagnosis, FIGO stage, tumour type, differentiation grade and residual tumour after primary surgery: 2) At least four of the following criteria: antigen retrieval, primary antibody, dilution, detection method, cut-off value for positive expression: 3) At least the primers used and the annealing temperature or number of cycles: 4) At least internal controls and probes used: 5) At least four of the following criteria: label, incubation time, filter size, separation method (BSA / Tris-sucrose), cut-off value for positive expression
