# Supplementary material for: Modest effect of p53, EGFR and HER-2/neu on prognosis in epithelial ovarian cancer: a meta-analysis
Source: Br J Cancer. 2009 Jun 9;101(1):149–59. doi: 10.1038/sj.bjc.6605112 (PMC2713689; doi:10.1038/sj.bjc.6605112)
Supplement: Supplementary Table 3 [file 6605112x3.doc]

***Supplementary table*** 3: Studies included in the meta-analysis for p53

| *Study* | *Year of publi- cation* | *Data collection* | *No. in study (of deaths)* | *Inclusion period* | *Specimen collection* | *Age in years* | *Stage* | *Tumour type* | *Assay*  *(antibody)* | *% positive tumours* | *Follow-up in months** | *Quality rating* |
| --- | --- | --- | --- | --- | --- | --- | --- | --- | --- | --- | --- | --- |
| [Allan L.A. et al., 1996] | 1996 | Prospective | 61 (42) | 1998-1993 | Europe | Median 63  (range 41 – 86) | All | All | IHC (Pab240, PAb 1801 and CM1) / sequencing | 61% | Range 0-68 | 6 |
| [Anttila et al., 1999] | 1999 | Retrospective | 316 (238) | 1996-1992 | Europe | - | All | All | IHC (CM1) | 27.1% | Median 29 (range 1 – 237) | 6 |
| [Baekelandt et al., 1999] | 1999 | Prospective | 185 (156) | 1988-1993 | Europe | Median 54  (range 21 – 70) | III | All | IHC (DO1) | 49% | Maximum 121 | 6 |
| [Bali et al., 2004] | 2004 | Retrospective | 134 | 1988-1998 | Australia | - | All | Serous | IHC (DO7) | 59% | Median 30 (range 3 – 136) | 6 |
| [Bartel et al., 2008] | 2008 | Retrospective | 107 | 1997-2005 | Europe | Median 64  Mean 63.5 | All | All | IHC (DO7) /  SSCP and sequencing | 51.7% (I)  39.2% (M) | - | 2 |
| [Berker et al., 2002] | 2002 | Retrospective | 50 (11) | 1990-1997 | Africa | Median 54 (range 25 - 71) | All | All | IHC | 66% | Median 45  (range 10 – 93) | 4 |
| [Birner et al., 2001] | 2001 | Retrospective | 102 (42) | - | Europe | Median 57 | All | All | IHC (DO7) | 56.9% | Mean 28  (range 1 – 130) | 5 |
| [Blegen et al., 2000] | 2000 | Retrospective | 52 | - | Europe | - | All | All | IHC (DO1) | 36.5% | - | 4 |
| [Brustmann, 2007] | 2007 | Retrospective | 50 (29) | 1985-2004 | Europe | Median 64  Mean 61.6  (range 30-81) | All | Serous | IHC (DO7) | 78% | - | 5 |
| [Ceccaroni et al., 2004] | 2004 | Retrospective | 52 (28) | 1986-1993 | Europe | - | All | All | IHC (BP53-12.1) | 49% | - | 1 |
| [Concin et al., 2005] | 2005 | Retrospective | 122 (60) | 1990-2001 | Europe | Median 61  (range 24-88) | All | All | Mutational analysis (yeast-based assay) | 65.6% | Median 55  (range 3-235) | 4 |
| [Darcy et al., 2008] | 2008 | Prospective | 143 /  (GOG-157);  136  (GOG-111)^4^ | - | North America | Median 58 /  Median 60 | I, II /  III, IV | All | IHC (DO7) | 51% /  62.% | Median 105  (range 14-137); median 127  (range 15-194) | 6 / 6 |
| [Darai et al., 1998] | 1997 | Retrospective | 20 (17) | - | Europe | Median 53.3 | All | All | IHC (DO7) | 65% | Mean 56  (range 11 – 100) | 4 |
| [Eltabbakh et al., 1997] | 1997 | Retrospective | 221 (108) | 1981-1994 | North America | Median 61 (range 29 – 90) | All | All | IHC | 48.4% | Maximum 168 | 5 |
| [Garcia-Velasco et al., 2008] | 2008 | Retrospective | 72 (21) | 1999-2003 | Europe | Median 57  (range 28-82) | - | All | IHC | 62.5% | Median 33  (range 1-193) | 4 |
| [Galic et al., 2007] | 2007 | Retrospective | 188 | - | North America | - | All | All | Sequencing | 57% | Range 1-154 | 4 |
| [Giordano et al., 2008] | 2008 | Retrospective | 52 (25) | 1989-2001 | Europe | - | - | All | IHC (DO7) | 26.9% | Maximum 132 | 5 |
| [Goodheart et al., 2005] | 2005 | Retrospective | 77 (16) | 1988-1999 | North America | Mean 50  (range 21 – 85) | I | All | IHC /  sequencing | 26% (I)  16% (M) | Maximum 176 | 3 / 3 ^3^ |
| [de Graeff et al., 2006]^5^ | 2006 | Prospective | 288 (200) | 1989-2003 | Europe | Median 58  (range 23-87) | All | All | IHC (DO7) | 53.8% | Median 44.3  (range 1-137) | 8 |
| [Green et al., 2006] | 2006 | Prospective | 169 (156) | 1987-1993 | Europe | Median 59.6  (range 32-83) | II-IV | All | IHC (DO1) | 92 (61%) | “> 9 years” | 6 |
| [Hartmann et al., 1994] | 1994 | Retrospective | 284 (184) | 1976-1990 | North America | Median 61  (range 19 – 86) | All | All | IHC (Pab1801) | 62% | Median 84 | 5 |
| [Havrilesky et al., 2003] | 2003 | Prospective | 125 (92) | - | - | Mean 60 | III / IV | All | IHC (DO1) / sequencing | 77% (M),  66% and 55% (I)^1^ | - | 8 / 7 ^3^ |
| [Hawes et al., 2002] | 2002 | Prospective | 31 (30) | - | - | Median 62  (range 30 – 73) | III / IV | All | IHC (Pab1801) | 48% | - | 6 |
| [Howells et al., 2001] | 2001 | Retrospective | 81 | 1990-1997 | Europe | Mean 61  (range 28 – 90) | All | All | IHC (DO7) | 42% | - | 4 |
| [Iba et al., 2004] | 2004 | Retrospective | 101(48) | 1996-2000 | Asia | Median 55  (range 21 – 82) | All | All | SSCP and sequencing | 50.5% | - | 4 |
| [Ikeda et al., 2003] | 2003 | Retrospective | 93 | 1990-2000 | Asia | Median 56  (range 26 – 77) | All | All | IHC (DO7) | 41.9% | - | 2 |
| [Kaern et al., 2005] | 2005 | Retrospective | 51 | 1990-1992 | Europe | Range 30 - 67 | III | All | IHC (DO1) | 82% | Range 5 - 159 | 4 |
| [Kaiser et al., 2005] | 2005 | Retrospective | 80 | 1984-1996 | Europe | Median 58  (range 18 – 82) | All | All | IHC (DO7) | 22.5% | - | 3 |
| [Kassim et al., 1999] | 1999 | Retrospective | 26 (12) | 1995-1995 | Africa | Mean 44  (range 25 – 66) | All | All | EIA | 57.7% | Mean 22  (range 9 – 37) | 2 |
| [Klemi et al., 1995] | 1995 | Retrospective | 136 (109) | 1963-1990 | Africa | Median 59  (range 29 – 79) | All | All | IHC | 44% | Maximum 292 | 6 |
| [Kobel et al., 2008] | 2008 | Retrospective | 500 (233) | 1984-200 | North America | Mean 58.1 | All | All | IHC (DO7) | 25.4% | Mean 70.8 | 5 |
| [Konstantinidou et al., 2003] | 2003 | Retrospective | 83 (22) | 1989-1999 | Europe | Median 53  (range 20-78) | All | All | IHC (DO1) | 47.6% | Range 1 - 126 | 6 |
| [Laframboise et al., 2000] | 2000 | Retrospective | 43 (18) | 1995-1997 | North America | Mean 57  (range 44 – 76) | II, III, IV | All | SSCP and sequencing | 53.5% | - | 4 |
| [Lee et al., 2006] | 2006 | Retrospective | 54 (34) | 1988-1998 | North America | - | All | All | IHC (DO7) | 64.8% | Median 67  (range 3-119) | 4 |
| [Leffers et al., 2008] | 2008 | Prospective | 329 (185) | 1985-2006 | Europe | Median 59  (range 16-89) | All | All | IHC (DO7) | 50% | - | 5 |
| [Levesque et al., 2000] | 2000 | Retrospective | 122 (44) | 1988-1997 | Europe | Mean 55  Median 55  (range 26 - 77) | All | All | EIA | 50.9% | Mean 30  Median 24  (range 3 – 119) | 5 |
| [Malamou-Mitsi et al., 2007] | 2007 | Prospective | 95 (62) | >1995 | Europe | Range 27-76 | All | All | IHC (DO1) | 29% | Median 66  (range 0.4-89) | 5 |
| [Marx et al., 1998] | 1998 | Retrospective | 187 (136) | 1982-1992 | Germany | - | All | All | IHC (DO7) | 14.4% | Median 22  (range 1-162) | 4 |
| [Materna et al., 2007] | 2007 | Retrospective | 43 (13) | 1999-2002 | Europe | Mean 51.0 | All | All | IHC (DO7) | 46.5% | - | 3 |
| [Nakayama et al., 2003] | 2003 | Retrospective | 134 (40) | - | Asia | Range 19 – 76 | All | All | IHC (DO7) | 25% | Median 47  (range 6 – 165) | 6 |
| [Nielsen et al., 2004] | 2004 | Prospective | 783 (610) | 1981-1986 and  1991-1994 | Europe | Median 58  (range 13 – 91) | All | All | IHC (DO7) | 53% | Median: 214 | 5 |
| [Ozalp et al., 2000] | 2000 | Retrospective | 26 (14) | - | Europe | Mean 51  (range 24 – 68) | All | All | IHC (DO7)/ FISH (P5107) | 46.1% (I)  26.9% (F) | - | 4 (I)  5 (F) |
| [Pieretti et al., 2002] | 2002 | Retrospective | 121 (52) | 1990-1996 | North America | Mean 58 | All | All | IHC (Pab1801) / SSCP and sequencing | 43% (I)  52% (M) | Median 29 | 2 / 2 ^3^ |
| [Psyrri et al., 2007] | 2007 | Retrospective | 141 | 1996-2003 | Europe | - | III, IV | All | IHC (DO7) | 81.6% (nuclear/  cytoplas-mic | Mean 34  (range 1-92) | 7 |
| [Reles et al., 2001] | 2001 | Retrospective | 178 (117) | 1972-1995 | Europe-and North America | Median 57  (range 23 – 84) | All | All | IHC (DO7) /  SSCP and sequencing | 62% (I)  56% (M) | Median 31  (range 1 –144) | 6 / 6 ^3^ |
| [Saegusa et al., 2001] | 2001 | Retrospective | 131 | 1992-2000 | Europe | Mean: 55  (range 28 - 82) | All | All | IHC (DO7) | 38.2% | Median 43  (range 1-110) | 4 |
| [Sagarra et al., 2002] | 2002 | Retrospective | 90 | 1990-1996 | South America | Median 53  (range 20 – 78) | All | All | IHC (DO7) | 47% | - | 5 |
| [Schildkraut et al., 2000] | 2000 | Prospective | 197 | 1980-1982 | North America | - | All | All | IHC (Pab1801) | 45.7% | - | 6 |
| [Schuyer et al., 2001] | 2001 | Retrospective | 102 | 1988-1993 | Europe | - | All | All | IHC (DO7) / SSCP and sequencing | 44% (I)  39% (M) | Maximum 120 | 5 / 3 ^3^ |
| [Seo et al., 2004] | 2004 | Retrospective | 64 (32) | 1992-1995 | Asia | Median 51  (range 18 – 75) | All | All | IHC | 40.6% | Median 56  (range 6 – 68) | 4 |
| [Shahin et al., 2000] | 2000 | Retrospective | 171 (100) | 1990-1996 | North America | Mean 58  (range 31 – 85) | All | All | IHC (DO7) /  SSCP and sequencing | 48.5% (I)  57.3%(M) | Median 41  (range 0 – 107) | 4 |
| [Silvestrini et al., 1998] | 1998 | Prospective | 168 | 1989-1994 | Europe | - | III, IV | All | IHC (Pab1801) | 67% (P)^2^  63% (PC) | Median 36  Minimum 6 | 8 |
| [Skirnisdottir et al., 2001] | 2001 | Retrospective | 107 (29) | 1988-1993 | Europe | Mean 60  (range 28 – 62) | I, II | All | IHC (DO7) | 21.7% | Median 87  (57 – 125) | 4 |
| [Terauchi et al., 2005] | 2005 | Retrospective | 43 (17) | 1990-2003 | Asia | - | All | Serous | IHC (DO7) | 49% | Median 63.7  (range 4 - 139) | 4 |
| [Tachibana et al., 2003] | 2003 | Retrospective | 73 | - | Asia | - | All | All | IHC (DO1, DO7, BP53-12) | 17.8% (a)  13.7% (b)  21.9% (c) | - | 2 |
| [Tomsova et al., 2008] | 2008 | Retrospective | 116 | 1996-2003 | Europe | Median 53  (range 27-82) | All | All | IHC (DO7) | 75.8% | Median 39  (range 1-120) | 4 |
| [Ueno et al., 2006] | 2006 | Prospective | 100 (48) | - | Asia | Median 58  (range 23-77) | All | All | Sequencing | 42% | Median 52  (range 17-93) | 8 |
| [Vartiainen et al., 2008] | 2008 | Retrospective | 173 (85) | 1990-2000 | Europe | - | All | Serous | IHC (DO7) | 62% | Median 39  (range 5-123) | 5 |
| [Viale et al., 1997] | 1997 | Retrospective | 112 | - | Europe | - | All | All | IHC (PAb1801) | 54.4% | Mean 46  (range 3 – 148) | 5 |
| [Wen et al., 1999] | 1999 | Retrospective | 105 (84) | - | North America | Median 56  (range 25 – 84) | All | All | IHC (DO7) | 68.8% | Median 29  (range 1 – 235) | 5 |
| [Wisman et al., 2003] | 2003 | Prospective | 47 (27) | 1988-1997 | Europe | - | All | - | IHC (BP53.12.1) | 68.3% | Median 42  Minimum 3 | 5 |
| [Yakirevich et al., 2006] | 2006 | Retrospective | 60 (32) | 1992-2002 | Other | Mean 62  (range 40-82) | All | Serous | IHC (BP53.12 | 75% | Median 42  (range 1-104) | 4 |

Abbreviations: I / IHC = immunohistochemistry; M = mutational analysis (SSCP and/or sequencing); EIA = enzyme immunoassay; FISH = Fluorescence in situ hybridization; a = DO1; b = DO7; c = BP53-12

1. Based on two different cut-off values for p53 expression: limited (>0% nuclear staining) and extensive (>30% nuclear staining), respectively
2. Results of this study were presented for the P arm (cisplatin treated patients) and the PC arm (cisplatin and cyclophosphamide treated patients) of the trial separately
3. Quality score for IHC staining / quality score for mutational analysis
4. Results for two different phase III clinical trials are reported: GOG-157 (three versus six cycles of paclitaxel/carboplatin in high-risk, early stage ovarian cancer) and GOG-111 (cyclophosphamide/cisplatin versus paclitaxel/cisplatin in suboptimally resected advanced stage ovarian cancer).
5. Reports results for a Dutch, hospital-based population and a prospective cohort of Scottish patients enrolled in clinical trials. As results for the Dutch cohort are more extensively described in Leffers et al [Leffers et al., 2008], only results for the Scottish cohort were included in the meta-analysis.

Reference List

Allan L.A., Campbell M.K., Milner B.J., Eccles D.M., Leonard R.C.F., Parkin D.E., Millers I.D., Lessells A.M., Kitchener H.C., Haites N.E. (1996) The significance of p53 mutation and over-expression in ovarian cancer prognosis. *Int J Gynecol Cancer* **6**: 483-490

Anttila MA, Ji H, Juhola MT, Saarikoski SV, Syrjanen KJ (1999) The prognostic significance of p53 expression quantitated by computerized image analysis in epithelial ovarian cancer. *Int J Gynecol Pathol* **18**: 42-51

Baekelandt M, Kristensen GB, Nesland JM, Trope CG, Holm R (1999) Clinical significance of apoptosis-related factors p53, Mdm2, and Bcl-2 in advanced ovarian cancer. *J Clin Oncol* **17**: 2061

Bali A, O'Brien PM, Edwards LS, Sutherland RL, Hacker NF, Henshall SM (2004) Cyclin D1, p53, and p21Waf1/Cip1 expression is predictive of poor clinical outcome in serous epithelial ovarian cancer. *Clin Cancer Res* **10**: 5168-5177

Bartel F, Jung J, Bohnke A, Gradhand E, Zeng K, Thomssen C, Hauptmann S (2008) Both germ line and somatic genetics of the p53 pathway affect ovarian cancer incidence and survival. *Clin Cancer Res* **14**: 89-96

Berker B, Dunder I, Ensari A, Cengiz SD (2002) Prognostic value of p53 accumulation in epithelial ovarian carcinomas. *Arch Gynecol Obstet* **266**: 205-209

Birner P, Schindl M, Obermair A, Breitenecker G, Oberhuber G (2001) Expression of hypoxia-inducible factor 1alpha in epithelial ovarian tumors: its impact on prognosis and on response to chemotherapy. *Clin Cancer Res* **7**: 1661-1668

Blegen H, Einhorn N, Sjovall K, Roschke A, Ghadimi BM, McShane LM, Nilsson B, Shah K, Ried T, Auer G (2000) Prognostic significance of cell cycle proteins and genomic instability in borderline, early and advanced stage ovarian carcinomas. *Int J Gynecol Cancer* **10**: 477-487

Brustmann H (2007) Poly(adenosine diphosphate-ribose) polymerase expression in serous ovarian carcinoma: correlation with p53, MIB-1, and outcome. *Int J Gynecol Pathol* **26**: 147-153

Ceccaroni M, Chieco P, Alboni C, De Laco P, Pagano K, Ceccarelli C, Santini D, Taroni B, Pelusi G (2004) p53 expression, DNA ploidy and mitotic index as prognostic factors in patients with epithelial ovarian carcinoma. *Tumori* **90**: 600-606

Concin N, Hofstetter G, Berger A, Gehmacher A, Reimer D, Watrowski R, Tong D, Schuster E, Hefler L, Heim K, Mueller-Holzner E, Marth C, Moll UM, Zeimet AG, Zeillinger R (2005) Clinical relevance of dominant-negative p73 isoforms for responsiveness to chemotherapy and survival in ovarian cancer: Evidence for a crucial p53-p73 cross-talk in vivo. *Clin Cancer Res* **11**: 8372-8383

Darai E, Walker-Combrouze F, Mlika-Cabanne N, Feldmann G, Madelenat P, Scoazec JY (1998) Expression of p53 protein in borderline epithelial ovarian tumors: a clinicopathologic study of 39 cases. *Eur J Gynaecol Oncol* **19**: 144-149

Darcy KM, Brady WE, McBroom JW, Bell JG, Young RC, McGuire WP, Linnoila RI, Hendricks D, Bonome T, Farley JH, Gynecologic Oncology Group (2008) Associations between p53 overexpression and multiple measures of clinical outcome in high-risk, early stage or suboptimally-resected, advanced stage epithelial ovarian cancers A Gynecologic Oncology Group study. *Gynecol Oncol* **111**: 487-495

de Graeff P, Hall J, Crijns AP, de Bock GH, Paul J, Oien KA, Ten Hoor KA, De Jong S, Hollema H, Bartlett JM, Brown R, Van Der Zee AG (2006) Factors influencing p53 expression in ovarian cancer as a biomarker of clinical outcome in multicentre studies. *Br J Cancer*

Eltabbakh GH, Belinson JL, Kennedy AW, Biscotti CV, Casey G, Tubbs RR, Blumenson LE (1997) p53 overexpression is not an independent prognostic factor for patients with primary ovarian epithelial cancer. *Cancer* **80**: 892-898

Galic V, Willner J, Wollan M, Garg R, Garcia R, Goff BA, Gray HJ, Swisher EM (2007) Common polymorphisms in TP53 and MDM2 and the relationship to TP53 mutations and clinical outcomes in women with ovarian and peritoneal carcinomas. *Genes, Chromosomes & Cancer* **46**: 239-247

Garcia-Velasco A, Mendiola C, Sanchez-Munoz A, Ballestin C, Colomer R, Cortes-Funes H (2008) Prognostic value of hormonal receptors, p53, ki67 and HER2/neu expression in epithelial ovarian carcinoma. *Clinical & Translational Oncology: Official Publication of the Federation of Spanish Oncology Societes & of the National Cancer Institute of Mexico* **10**: 367-371

Giordano G, Azzoni C, D'Adda T, Rocco A, Gnetti L, Froio E, Merisio C, Melpignano M (2008) Human papilloma virus (HPV) status, p16INK4a, and p53 overexpression in epithelial malignant and borderline ovarian neoplasms. *Pathology, Research & Practice* **204**: 163-174

Goodheart MJ, Ritchie JM, Rose SL, Fruehauf JP, De Young BR, Buller RE (2005) The relationship of molecular markers of p53 function and angiogenesis to prognosis of stage I epithelial ovarian cancer. *Clin Cancer Res* **11**: 3733-3742

Green JA, Berns EMJJ, Coens C, van Luijk I, Thompson-Hehir J, van Diest P, Verheijen RHM, van de Vijver M, van Dam P, Kenter GG, Tjalma W, Ewing PC, Teodorovic I, Vergote I, van der Burg MEL (2006) Alterations in the p53 pathway and prognosis in advanced ovarian cancer: A multi-factorial analysis of the EORTC Gynaecological Cancer group (study 55865). *Eur J Cancer* **42**: 2539-2548

Hartmann LC, Podratz KC, Keeney GL, Kamel NA, Edmonson JH, Grill JP, Su JQ, Katzmann JA, Roche PC (1994) Prognostic significance of p53 immunostaining in epithelial ovarian cancer. *J Clin Oncol* **12**: 64-69

Havrilesky L, Darcy M, Hamdan H, Priore RL, Leon J, Bell J, Berchuck A (2003) Prognostic significance of p53 mutation and p53 overexpression in advanced epithelial ovarian cancer: a Gynecologic Oncology Group Study. *J Clin Oncol* **21**: 3814-3825

Hawes D, Liu PY, Muggia FM, Wilczynski S, Cote R, Felix J, Terada K, Belt RJ, Alberts DS (2002) Correlation of p53 immunostaining in primary and residual ovarian cancer at the time of positive second-look laparotomy and its prognostic role: a Southwest Oncology Group ancillary study. *Gynecol Oncol* **87**: 17-23

Howells RE, Holland T, Dhar KK, Redman CW, Hand P, Hoban PR, Jones PW, Fryer AA, Strange RC (2001) Glutathione S-transferase GSTM1 and GSTT1 genotypes in ovarian cancer: association with p53 expression and survival. *Int J Gynecol Cancer* **11**: 107-112

Iba T, Kigawa J, Kanamori Y, Itamochi H, Oishi T, Simada M, Uegaki K, Naniwa J, Terakawa N (2004) Expression of the c-myc gene as a predictor of chemotherapy response and a prognostic factor in patients with ovarian cancer. *Cancer Sci* **95**: 418-423

Ikeda K, Sakai K, Yamamoto R, Hareyama H, Tsumura N, Watari H, Shimizu M, Minakami H, Sakuragi N (2003) Multivariate analysis for prognostic significance of histologic subtype, GST-pi, MDR-1, and p53 in stages II-IV ovarian cancer. *Int J Gynecol Cancer* **13**: 776-784

Kaern J, Aghmesheh M, Nesland JM, Danielsen HE, Sandstad B, Friedlander M, Trope C (2005) Prognostic factors in ovarian carcinoma stage III patients. Can biomarkers improve the prediction of short- and long-term survivors? *Int J Gynecol Cancer* **15**: 1014-1022

Kaiser PC, Korner M, Kappeler A, Aebi S (2005) Retinoid receptors in ovarian cancer: expression and prognosis. *Ann Oncol* **16**: 1477-1487

Kassim SK, Ali HS, Sallam MM, Fayed ST, Seada LS, abd-Elkawy E, Seada MA, Khalifa A (1999) Increased bcl-2 expression is associated with primary resistance to chemotherapy in human epithelial ovarian cancer. *Clin Biochem* **32**: 333-338

Klemi PJ, Pylkkanen L, Kiilholma P, Kurvinen K, Joensuu H (1995) p53 protein detected by immunohistochemistry as a prognostic factor in patients with epithelial ovarian carcinoma. *Cancer* **76**: 1201-1208

Kobel M, Kalloger SE, Boyd N, McKinney S, Mehl E, Palmer C, Leung S, Bowen NJ, Ionescu DN, Rajput A, Prentice LM, Miller D, Santos J, Swenerton K, Gilks CB, Huntsman D (2008) Ovarian carcinoma subtypes are different diseases: Implications for biomarker studies. *PLoS Med* **5**: 1749-1760

Konstantinidou AE, Korkolopoulou P, Vassilopoulos I, Tsenga A, Thymara I, Agapitos E, Patsouris E, Davaris P (2003) Reduced retinoblastoma gene protein to Ki-67 ratio is an adverse prognostic indicator for ovarian adenocarcinoma patients. *Gynecol Oncol* **88**: 369-378

Laframboise S, Chapman W, McLaughlin J, Andrulis IL (2000) p53 mutations in epithelial ovarian cancers: possible role in predicting chemoresistance. *Cancer J* **6**: 302-308

Lee JS, Choi YD, Lee JH, Nam JH, Choi C, Lee MC, Park CS, Juhng SW, Min KW (2006) Expression of cyclooxygenase-2 in epithelial ovarian tumors and its relation to vascular endothelial growth factor and p53 expression. *International Journal of Gynecological Cancer* **16**: Suppl-53

Leffers N, Lambeck AJ, de GP, Bijlsma AY, Daemen T, Van Der Zee AG, Nijman HW (2008) Survival of ovarian cancer patients overexpressing the tumour antigen p53 is diminished in case of MHC class I down-regulation. *Gynecol Oncol* **110**: 365-373

Levesque MA, Katsaros D, Massobrio M, Genta F, Yu H, Richiardi G, Fracchioli S, Durando A, Arisio R, Diamandis EP (2000) Evidence for a dose-response effect between p53 (but not p21WAF1/Cip1) protein concentrations, survival, and responsiveness in patients with epithelial ovarian cancer treated with platinum-based chemotherapy. *Clin Cancer Res* **6**: 3260-3270

Malamou-Mitsi V, Crikoni O, Timotheadou E, Aravantinos G, Vrettou E, Agnantis N, Fountzilas G (2007) Prognostic significance of HER-2, p53 and Bcl-2 in patients with epithelial ovarian cancer. *Anticancer Research* **27**: 1157-1165

Marx D, Meden H, Ziemek T, Lenthe T, Kuhn W, Schauer A (1998) Expression of the p53 tumour suppressor gene as a prognostic marker in platinum-treated patients with ovarian cancer. *Eur J Cancer* **34**: 845-850

Materna V, Surowiak P, Markwitz E, Spaczynski M, Drag-Zalesinska M, Zabel M, Lage H (2007) Expression of factors involved in regulation of DNA mismatch repair- and apoptosis pathways in ovarian cancer patients. *Oncology Reports* **17**: 505-516

Nakayama K, Takebayashi Y, Nakayama S, Hata K, Fujiwaki R, Fukumoto M, Miyazaki K (2003) Prognostic value of overexpression of p53 in human ovarian carcinoma patients receiving cisplatin. *Cancer Lett* **192**: 227-235

Nielsen JS, Jakobsen E, Holund B, Bertelsen K, Jakobsen A (2004) Prognostic significance of p53, Her-2, and EGFR overexpression in borderline and epithelial ovarian cancer. *Int J Gynecol Cancer* **14**: 1086-1096

Ozalp SS, Yalcin OT, Basaran GN, Artan S, Kabukcuoglu S, Minsin TH (2000) Prognostic significance of deletion and over-expression of the p53 gene in epithelial ovarian cancer. *Eur J Gynaecol Oncol* **21**: 282-286

Pieretti M, Hopenhayn-Rich C, Khattar NH, Cao Y, Huang B, Tucker TC (2002) Heterogeneity of ovarian cancer: relationships among histological group, stage of disease, tumor markers, patient characteristics, and survival. *Cancer Invest* **20**: 11-23

Psyrri A, Kountourakis P, Yu Z, Papadimitriou C, Markakis S, Camp RL, Economopoulos T, Dimopoulos MA (2007) Analysis of p53 protein expression levels on ovarian cancer tissue microarray using automated quantitative analysis elucidates prognostic patient subsets. *Annals of Oncology* **18**: 709-715

Reles A, Wen WH, Schmider A, Gee C, Runnebaum IB, Kilian U, Jones LA, El Naggar A, Minguillon C, Schonborn I, Reich O, Kreienberg R, Lichtenegger W, Press MF (2001) Correlation of p53 mutations with resistance to platinum-based chemotherapy and shortened survival in ovarian cancer. *Clin Cancer Res* **7**: 2984-2997

Saegusa M, Machida BD, Okayasu I (2001) Possible associations among expression of p14(ARF), p16(INK4a), p21(WAF1/CIP1), p27(KIP1), and p53 accumulation and the balance of apoptosis and cell proliferation in ovarian carcinomas. *Cancer* **92**: 1177-1189

Sagarra RA, Andrade LA, Martinez EZ, Pinto GA, Syrjanen KJ, Derchain SF (2002) P53 and Bcl-2 as prognostic predictors in epithelial ovarian cancer. *Int J Gynecol Cancer* **12**: 720-727

Schildkraut JM, Halabi S, Bastos E, Marchbanks PA, McDonald JA, Berchuck A (2000) Prognostic factors in early-onset epithelial ovarian cancer: a population-based study. *Obstet Gynecol* **95**: 119-127

Schuyer M, van der Burg ME, Henzen-Logmans SC, Fieret JH, Klijn JG, Look MP, Foekens JA, Stoter G, Berns EM (2001) Reduced expression of BAX is associated with poor prognosis in patients with epithelial ovarian cancer: a multifactorial analysis of TP53, p21, BAX and BCL-2. *Br J Cancer* **85**: 1359-1367

Seo SS, Song YS, Kang DH, Park IA, Bang YJ, Kang SB, Lee HP (2004) Expression of cyclooxygenase-2 in association with clinicopathological prognostic factors and molecular markers in epithelial ovarian cancer. *Gynecol Oncol* **92**: 927-935

Shahin MS, Hughes JH, Sood AK, Buller RE (2000) The prognostic significance of p53 tumor suppressor gene alterations in ovarian carcinoma. *Cancer* **89**: 2006-2017

Silvestrini R, Daidone MG, Veneroni S, Benini E, Scarfone G, Zanaboni F, Villa A, Presti M, Danese S, Bolis G (1998) The clinical predictivity of biomarkers of stage III-IV epithelial ovarian cancer in a prospective randomized treatment protocol. *Cancer* **82**: 159-167

Skirnisdottir I, Sorbe B, Seidal T (2001) P53, bcl-2, and bax: their relationship and effect on prognosis in early stage epithelial ovarian carcinoma. *Int J Gynecol Cancer* **11**: 147-158

Tachibana M, Watanabe J, Matsushima Y, Nishida K, Kobayashi Y, Fujimura M, Shiromizu K (2003) Independence of the prognostic value of tumor suppressor protein expression in ovarian adenocarcinomas: A multivariate analysis of expression of p53, retinoblastoma, and related proteins. *Int J Gynecol Cancer* **13**: 598-606

Terauchi F, Okamoto A, Nagashima T, Kobayashi Y, Moritake T, Yamamoto Y, Takakura S, Iwaki S, Ogura H (2005) Clinical significance of p21(WAF1/CIP1) and p53 expression in serous cystadenocarcinoma of the ovary. *Oncol Rep* **14**: 363-368

Tomsova M, Melichar B, Sedlakova I, Steiner I (2008) Prognostic significance of CD3+ tumor-infiltrating lymphocytes in ovarian carcinoma. *Gynecol Oncol* **108**: 415-420

Ueno Y, Enomoto T, Otsuki Y, Sugita N, Nakashima R, Yoshino K, Kuragaki C, Ueda Y, Aki T, Ikegami H, Yamazaki M, Ito K, Nagamatsu M, Nishizaki T, Asada M, Kameda T, Wakimoto A, Mizutani T, Yamada T, Murata Y (2006) Prognostic significance of p53 mutation in suboptimally resected advanced ovarian carcinoma treated with the combination chemotherapy of paclitaxel and carboplatin. *Cancer Lett* **241**: 289-300

Vartiainen J, Lassus H, Lehtovirta P, Finne P, Alfthan H, Butzow R, Stenman UH (2008) Combination of serum hCG beta and p53 tissue expression defines distinct subgroups of serous ovarian carcinoma. *International Journal of Cancer* **122**: 2125-2129

Viale G, Maisonneuve P, Bonoldi E, Di Bacco A, Bevilacqua P, Panizzoni GA, Radaelli U, Gasparini G (1997) The combined evaluation of p53 accumulation and of Ki-67 (MIB1) labelling index provides independent information on overall survival of ovarian carcinoma patients. *Ann Oncol* **8**: 469-476

Wen WH, Reles A, Runnebaum IB, Sullivan-Halley J, Bernstein L, Jones LA, Felix JC, Kreienberg R, El Naggar A, Press MF (1999) p53 mutations and expression in ovarian cancers: correlation with overall survival. *Int J Gynecol Pathol* **18**: 29-41

Wisman GB, Hollema H, Helder MN, Knol AJ, Van der Meer GT, Krans M, De Jong S, De Vries EG, Van Der Zee AG (2003) Telomerase in relation to expression of p53, c-Myc and estrogen receptor in ovarian tumours. *Int J Oncol* **23**: 1451-1459

Yakirevich E, Sabo E, Naroditsky I, Sova Y, Lavie O, Resnick MB (2006) Multidrug resistance-related phenotype and apoptosis-related protein expression in ovarian serous carcinomas. *Gynecol Oncol* **100**: 152-159
