# Supplementary material for: Modest effect of p53, EGFR and HER-2/neu on prognosis in epithelial ovarian cancer: a meta-analysis
Source: Br J Cancer. 2009 Jun 9;101(1):149–59. doi: 10.1038/sj.bjc.6605112 (PMC2713689; doi:10.1038/sj.bjc.6605112)
Supplement: Supplementary Table 4 [file 6605112x4.doc]

## Supplementary table 4: Studies included in the meta-analysis for EGFR

| *Study* | *Year of publi- cation* | *Data collection* | *No. in study (of deaths)* | *Inclusion period* | *Specimen collection* | *Age in years* | *Stage* | *Tumour type* | *Assay*  *(antibody)* | *% positive tumours* | *Follow-up in months* | *Quality rating* |
| --- | --- | --- | --- | --- | --- | --- | --- | --- | --- | --- | --- | --- |
| [Bartlett et al., 1996] | 1996 | Retrospective | 62 (33) | - | Europe | - | All | All | RT-PCR | 70% | - | 3 |
| [Brustmann, 2008] | 2008 | Retrospective | 50 (29) | 1985-2004 | Europe | Median 64  (range 30-81) | All | Serous | IHC (NCL-EGFR-384) | 26% | - | 5 |
| [Castellvi et al., 2006] | 2006 | Prospective | 75 (27) | 1994-1999 | Europe | - | All | All | IHC | 10.7% | - | 3 |
| [Elie et al., 2004] | 2004 | Prospective | 93 (74) | 1994-1997 | Europe | Median 60  Range 23 - 70 | III-IV | All | IHC (EGFR.113) | 33.3% | Median 69 | 7 |
| [Fischer-Colbrie et al., 1997] | 1997 | Retrospective | 108 (47) | 1993-1998 | Europe | Range 25 - 85 | All | All | [ ^125^I ] EGF binding assay | 61% | Mean 50.7 | 4 |
| [de Graeff et al., 2008] | 2008 | Prospective | 232 | 1985-2002 | Europe | Median 57.8  (range 22-90) | All | All | IHC (31G7) | 6.2% | - | 5 |
| [Kaufmann et al., 1995] | 1995 | Retrospective | 77 (43) | 1984-1990 | Europe | Median 63  (range 33-83) | All | All | [ ^125^I ] EGF binding assay | 66% | Median 19  (range 4 – 89) | 3 |
| [Lassus et al., 2006] | 2006 | Retrospective | 398 (184) | 1980-2000 | Europe | - | All | Serous | IHC  (NCL-EGFR) | 17.5% | Median 60  (range 0.4-248) | 6 |
| [Nielsen et al., 2004] | 2004 | Prospective | 783 (610) | 1981-1986 and  1991-1994 | Europe | Median 58  (range 13 – 91) | All | All | IHC (EGFR.113) | 62% | Median: 214 | 5 |
| [Psyrri et al., 2005] | 2005 | Retrospective | 81 (29) | 1996-2003 | Europe | Median 59 | All | All | IHC (H11) | 16% | Mean 34.4 (range 1 - 91.7) | 7 |
| [Raspollini et al., 2005] | 2005 | Retrospective | 60 | 1985-1992 | Europe | Median 58  (range 33 – 75) | III | Serous | IHC (31G7) | 23.3% | - | 3 |
| [Scambia et al., 1995] | 1995 | Retrospective | 117 (45) | - | Europe | - | All | All | [ ^125^I ] EGF binding assay | 54% | Median 19  (range 2 – 110) | 5 |
| [Schilder et al., 2005] | 2005 | Prospective | 27 (21) | - | U.S. | Median 61  (range 34 – 83) | - | All | IHC (monoclonal ab, Zymed) | 42% | - | 5 |
| [Skirnisdottir et al., 2001] | 2001 | Retrospective | 106 (29) | 1988-1993 | Europe | Mean 60  (range 26 – 81) | I-II | All | IHC (EGFR.113) | 34.9% | Median 87  (range 57 – 125) | 5 |
| [Wang et al., 2005] | 2005 | Retrospective | 118 (90) | 1992-2003 | Europe | Median 60  (range 38 – 81) | All | All | IHC (H11) | 55.9% | Maximum 142 | 4 |

Abbreviations: I / IHC = immunohistochemistry; M = mutational analysis (SSCP and/or sequencing); EIA = enzyme immunoassay; FISH = Fluorescence in situ hybridization

Reference List

Bartlett JM, Langdon SP, Simpson BJ, Stewart M, Katsaros D, Sismondi P, Love S, Scott WN, Williams AR, Lessells AM, Macleod KG, Smyth JF, Miller WR (1996) The prognostic value of epidermal growth factor receptor mRNA expression in primary ovarian cancer. *Br J Cancer* **73**: 301-306

Brustmann H (2008) Epidermal growth factor receptor expression in serous ovarian carcinoma: an immunohistochemical study with galectin-3 and cyclin D1 and outcome. *Int J Gynecol Pathol* **27**: 380-389

Castellvi J, Garcia A, Rojo F, Ruiz-Marcellan C, Gil A, Baselga J, Ramon YC (2006) Phosphorylated 4E binding protein 1: A hallmark of cell signaling that correlates with survival in ovarian cancer. *Cancer* **107**: 1801-1811

de Graeff P, Crijns AP, Ten Hoor KA, Klip HG, Hollema H, Oien K, Bartlett JM, Wisman GB, de Bock GH, De Vries EG, De Jong S, Van Der Zee AG (2008) The ErbB signalling pathway: protein expression and prognostic value in epithelial ovarian cancer. *Br J Cancer* **99**: 341-349

Elie C, Geay JF, Morcos M, Le Tourneau A, Girre V, Broet P, Marmey B, Chauvenet L, Audouin J, Pujade-Lauraine E, Camilleri-Broet S (2004) Lack of relationship between EGFR-1 immunohistochemical expression and prognosis in a multicentre clinical trial of 93 patients with advanced primary ovarian epithelial cancer (GINECO group). *Br J Cancer* **91**: 470-475

Fischer-Colbrie J, Witt A, Heinzl H, Speiser P, Czerwenka K, Sevelda P, Zeillinger R (1997) EGFR and steroid receptors in ovarian carcinoma: comparison with prognostic parameters and outcome of patients. *Anticancer Res* **17**: 613-619

Kaufmann M, Von Minckwitz G, Kuhn W, Schmid H, Costa S, Goerttler K, Bastert G (1995) Combination of new biologic parameters as a prognostic index in epithelial ovarian carcinoma. *Int J Gynecol Cancer* **5**: 49-55

Lassus H, Sihto H, Leminen A, Joensuu H, Isola J, Nupponen NN, Butzow R (2006) Gene amplification, mutation, and protein expression of EGFR and mutations of ERBB2 in serous ovarian carcinoma. *J Mol Med*

Nielsen JS, Jakobsen E, Holund B, Bertelsen K, Jakobsen A (2004) Prognostic significance of p53, Her-2, and EGFR overexpression in borderline and epithelial ovarian cancer. *Int J Gynecol Cancer* **14**: 1086-1096

Psyrri A, Kassar M, Yu Z, Bamias A, Weinberger PM, Markakis S, Kowalski D, Camp RL, Rimm DL, Dimopoulos MA (2005) Effect of epidermal growth factor receptor expression level on survival in patients with epithelial ovarian cancer. *Clin Cancer Res* **11**: 8637-8643

Raspollini MR, Castiglione F, Garbini F, Villanucci A, Amunni G, Baroni G, Boddi V, Taddei GL (2005) Correlation of epidermal growth factor receptor expression with tumor microdensity vessels and with vascular endothelial growth factor expression in ovarian carcinoma. *Int J Surg Pathol* **13**: 135-142

Scambia G, Benedetti-Panici P, Ferrandina G, Distefano M, Salerno G, Romanini ME, Fagotti A, Mancuso S (1995) Epidermal growth factor, oestrogen and progesterone receptor expression in primary ovarian cancer: correlation with clinical outcome and response to chemotherapy. *Br J Cancer* **72**: 361-366

Schilder RJ, Sill MW, Chen X, Darcy KM, Decesare SL, Lewandowski G, Lee RB, Arciero CA, Wu H, Godwin AK (2005) Phase II study of gefitinib in patients with relapsed or persistent ovarian or primary peritoneal carcinoma and evaluation of epidermal growth factor receptor mutations and immunohistochemical expression: a Gynecologic Oncology Group Study. *Clin Cancer Res* **11**: 5539-5548

Skirnisdottir I, Sorbe B, Seidal T (2001) The growth factor receptors HER-2/neu and EGFR, their relationship, and their effects on the prognosis in early stage (FIGO I-II) epithelial ovarian carcinoma. *Int J Gynecol Cancer* **11**: 119-129

Wang Y, Kristensen GB, Helland A, Nesland JM, Borresen-Dale AL, Holm R (2005) Protein expression and prognostic value of genes in the erb-b signaling pathway in advanced ovarian carcinomas. *Am J Clin Pathol* **124**: 392-401
