# Supplementary material for: Modest effect of p53, EGFR and HER-2/neu on prognosis in epithelial ovarian cancer: a meta-analysis
Source: Br J Cancer. 2009 Jun 9;101(1):149–59. doi: 10.1038/sj.bjc.6605112 (PMC2713689; doi:10.1038/sj.bjc.6605112)
Supplement: Supplementary Table 5 [file 6605112x5.doc]

## Supplementary table 5: Studies included in the meta-analysis for HER-2/neu

| *Study* | *Year of publi- cation* | *Data collection* | *No. in study (of deaths)* | *Inclusion period* | *Specimen collection* | *Age in years* | *Stage* | *Tumour type* | *Assay*  *(antibody)* | *% positive tumours* | *Follow-up in months* | *Quality score* |
| --- | --- | --- | --- | --- | --- | --- | --- | --- | --- | --- | --- | --- |
| [Berchuck et al., 1990] | 1990 | Retrospective | 73 (72) | 1985-1989 | US | - | III-IV | All | IHC (TA1) | 32% | - | 4 |
| [Camilleri-Broet et al., 2004] | 2004 | Prospective | 117 (88) | 1994-1997 | - | Median 59  (range 23 - 70) | All | All | IHC (CB11) | 16% | Median: 68 | 7 |
| [Castellvi et al., 2006] | 2006 | Prospective | 75 (27) | 1994-1999 | Europe | - | All | All | IHC | 30.7% | - | 3 |
| [Davidson et al., 2000] | 2000 | Retrospective | 45 (26) | 1977-1997 | Israel | Range 30 – 84 | III-IV | All | IHC | 57% | Mean: 70  (range 8 – 224) | 4 |
| [Fajac et al., 1995]**^1^** | 1995 | Retrospective | 65 (37) | 1984-1992 | Europe | - | All | All | Southern blot | 14% | Median: 71  (range 10 - 143) | 5 |
| [Garcia-Velasco et al., 2008] | 2008 | Retrospective | 72 (21) | 1999-2003 | Europe | Median 57  (range 28-82) | - | All | IHC (Herceptest) and FISH | 5% | Median 33  (range 1-193) | 4 |
| [de Graeff et al., 2008] | 2008 | Prospective | 232 | 1985-2002 | Europe | Median 57.8  (range 22-90) | All | All | IHC (NCL-CBE-356) | 5.1% | - | 5 |
| [Kaufmann et al., 1995] | 1995 | Retrospective | 77 | 1984-1990 | Europe | Median 63  (range 33 – 83) | All | All | Immunoassay | 29% | Median: 19  (range 4 – 89) | 3 |
| [Malamou-Mitsi et al., 2007] | 2007 | Prospective | 95 (62) | >1995 | Europe | Range 27-76 | All | All | IHC (MCO102) | 18% | Median 66  (range 0.4-89) | 5 |
| [Medl et al., 1995] | 1995 | Retrospective | 196 (118) | 1981-1989 | Europe | Median 59.6  (range 15-88) | All | All | PCR | 40.3% | Mean: 59 | 6 |
| [Nielsen et al., 2004] ^3^ | 2004 | Prospective | 783 (610) | 1981-1986 and  1991-1994 | Europe | Median 58  (range 13 – 91) | All | All | IHC (polyclonal rabbit ab, DAKO) | 35% | Median: 214 | 5 |
| [Pils et al., 2007] | 2007 | Retrospective | 128 (39) | - | Europe | Mean 59.2  (SD 12.1) | All | All | IHC (Herceptest) | 27.6% | Median 43.7  (range -.4-169) | 5 |
| [Skirnisdottir et al., 2001] | 2001 | Retrospective | 106 (29) | 1988-1993 | Europe | Mean 60  (range 26 – 82) | I-II | All | IHC (polyclonal rabbit ab, DAKO) | 18.9% | Median: 87  (range 57 – 125) | 5 |
| [Steffensen et al., 2007] | 2007 | Prospective | 160 (134) | 1991-1994 | Europe | Median 54.5  (range 29-70) | All | All | IHC (Herceptest)  and FISH | 35.6% (I)  6.9% (F)  6.3% (I/F) | - | 8 |
| [Surowiak et al., 2006] | 2006 | Retrospective | 43 (13) | 1999-2002 | Europe | Mean 51 | All | All | IHC | 51.2% | Range 0-52 | 5 |
| [Tomsova et al., 2008] | 2008 | Retrospective | 116 | 1996-2003 | Europe | Median 53  (range 27-82) | All | All | IHC (Herceptest) | 8.6% | Median 39  (range 1-120) | 4 |
| [Tuefferd et al., 2007] | 2007 | Prospective | 320 (66) | 2002-2004 | - | Median 58  (range 25-77) | All | All | IHC (CB11) and FISH | 6.6% | Median 24.9 | 6 |
| [Verri et al., 2005] | 2005 | Retrospective | 194 | 1990-2002 | Europe | Median 57  Range 25 – 90 | All | All | IHC (Herceptest) | 13.9% | Median: 45  (range 1 – 161) | 4 |
| [Wang et al., 2005] | 2005 | Retrospective | 118 | 1992-2003 | Europe | Median 60  Range 31 – 81 | II - IV | All | IHC (CB11) | 15.3% | Maximum 142 | 4 |
| [Wang et al., 1999] | 1999 | Retrospective | 40(23) | 1993-1995 | USA | Mean 59.2  Median 61  Range 35 - 83 | All | All | FISH | 25% | Range 1 - 56 | 3 |

Abbreviations: I / IHC = immunohistochemistry; P =polymerase chain reaction; ab = antibody; F / FISH = Fluorescence in situ hybridization; SD = standard deviation

1. Results for her-2/neu gene amplification; 2. Results for stage III/IV patients only; 3. These results indicate that her-2/neu expression is associated with increased survival

Reference List

Berchuck A, Kamel A, Whitaker R, Kerns B, Olt G, Kinney R, Soper JT, Dodge R, Clarke-Pearson DL, Marks P, . (1990) Overexpression of HER-2/neu is associated with poor survival in advanced epithelial ovarian cancer. *Cancer Res* **50**: 4087-4091

Camilleri-Broet S, Hardy-Bessard AC, Le Tourneau A, Paraiso D, Levrel O, Leduc B, Bain S, Orfeuvre H, Audouin J, Pujade-Lauraine E (2004) HER-2 overexpression is an independent marker of poor prognosis of advanced primary ovarian carcinoma: a multicenter study of the GINECO group. *Ann Oncol* **15**: 104-112

Castellvi J, Garcia A, Rojo F, Ruiz-Marcellan C, Gil A, Baselga J, Ramon YC (2006) Phosphorylated 4E binding protein 1: A hallmark of cell signaling that correlates with survival in ovarian cancer. *Cancer* **107**: 1801-1811

Davidson B, Gotlieb WH, Ben Baruch G, Nesland JM, Bryne M, Goldberg I, Kopolovic J, Berner A (2000) E-Cadherin complex protein expression and survival in ovarian carcinoma. *Gynecol Oncol* **79**: 362-371

de Graeff P, Crijns AP, Ten Hoor KA, Klip HG, Hollema H, Oien K, Bartlett JM, Wisman GB, de Bock GH, De Vries EG, De Jong S, Van Der Zee AG (2008) The ErbB signalling pathway: protein expression and prognostic value in epithelial ovarian cancer. *Br J Cancer* **99**: 341-349

Fajac A, Benard J, Lhomme C, Rey A, Duvillard P, Rochard F, Bernaudin JF, Riou G (1995) c-erbB2 gene amplification and protein expression in ovarian epithelial tumors: evaluation of their respective prognostic significance by multivariate analysis. *Int J Cancer* **64**: 146-151

Garcia-Velasco A, Mendiola C, Sanchez-Munoz A, Ballestin C, Colomer R, Cortes-Funes H (2008) Prognostic value of hormonal receptors, p53, ki67 and HER2/neu expression in epithelial ovarian carcinoma. *Clinical & Translational Oncology: Official Publication of the Federation of Spanish Oncology Societes & of the National Cancer Institute of Mexico* **10**: 367-371

Kaufmann M, Von Minckwitz G, Kuhn W, Schmid H, Costa S, Goerttler K, Bastert G (1995) Combination of new biologic parameters as a prognostic index in epithelial ovarian carcinoma. *Int J Gynecol Cancer* **5**: 49-55

Malamou-Mitsi V, Crikoni O, Timotheadou E, Aravantinos G, Vrettou E, Agnantis N, Fountzilas G (2007) Prognostic significance of HER-2, p53 and Bcl-2 in patients with epithelial ovarian cancer. *Anticancer Research* **27**: 1157-1165

Medl M, Sevelda P, Czerwenka K, Dobianer K, Hanak H, Hruza C, Klein M, Leodolter S, Mullauer-Ertl S, Rosen A, . (1995) DNA amplification of HER-2/neu and INT-2 oncogenes in epithelial ovarian cancer. *Gynecol Oncol* **59**: 321-326

Nielsen JS, Jakobsen E, Holund B, Bertelsen K, Jakobsen A (2004) Prognostic significance of p53, Her-2, and EGFR overexpression in borderline and epithelial ovarian cancer. *Int J Gynecol Cancer* **14**: 1086-1096

Pils D, Pinter A, Reibenwein J, Alfanz A, Horak P, Schmid BC, Hefler L, Horvat R, Reinthaller A, Zeillinger R, Krainer M (2007) In ovarian cancer the prognostic influence of HER2/neu is not dependent on the CXCR4/SDF-1 signalling pathway. *Br J Cancer* **96**: 485-491

Skirnisdottir I, Sorbe B, Seidal T (2001) The growth factor receptors HER-2/neu and EGFR, their relationship, and their effects on the prognosis in early stage (FIGO I-II) epithelial ovarian carcinoma. *Int J Gynecol Cancer* **11**: 119-129

Steffensen KD, Waldstrom M, Jeppesen U, Jakobsen E, Brandslund I, Jakobsen A (2007) The prognostic importance of cyclooxygenase 2 and HER2 expression in epithelial ovarian cancer. *International Journal of Gynecological Cancer* **17**: 798-807

Surowiak P, Materna V, Kaplenko I, Spaczynski M, Dietel M, Lage H, Zabel M (2006) Topoisomerase 1A, HER/2neu and Ki67 expression in paired primary and relapse ovarian cancer tissue samples. *Histology & Histopathology* **21**: 713-720

Tomsova M, Melichar B, Sedlakova I, Steiner I (2008) Prognostic significance of CD3+ tumor-infiltrating lymphocytes in ovarian carcinoma. *Gynecol Oncol* **108**: 415-420

Tuefferd M, Couturier J, Penault-Llorca F, Vincent-Salomon A, Broet P, Guastalla JP, Allouache D, Combe M, Weber B, Pujade-Lauraine E, Camilleri-Broet S (2007) HER2 status in ovarian carcinomas: A multicenter GINECO study of 320 patients. *PLoS ONE* **2**:

Verri E, Guglielmini P, Puntoni M, Perdelli L, Papadia A, Lorenzi P, Rubagotti A, Ragni N, Boccardo F (2005) HER2/neu oncoprotein overexpression in epithelial ovarian cancer: evaluation of its prevalence and prognostic significance. Clinical study. *Oncology* **68**: 154-161

Wang Y, Kristensen GB, Helland A, Nesland JM, Borresen-Dale AL, Holm R (2005) Protein expression and prognostic value of genes in the erb-b signaling pathway in advanced ovarian carcinomas. *Am J Clin Pathol* **124**: 392-401

Wang ZR, Liu W, Smith ST, Parrish RS, Young SR (1999) c-myc and chromosome 8 centromere studies of ovarian cancer by interphase FISH. *Exp Mol Pathol* **66**: 140-148
